# Lysosome-associated membrane glycoprotein 3 is involved in influenza A virus replication in human lung epithelial (A549) cells

**DOI:** 10.1186/1743-422X-8-384

**Published:** 2011-08-03

**Authors:** Zhuo Zhou, Qinghua Xue, Yuli Wan, Yaowu Yang, Jianwei Wang, Tao Hung

**Affiliations:** 1 State Key Laboratory of Molecular Virology and Genetic Engineering, Institute of Pathogen Biology, Peking Union Medical College & Chinese Academy of Medical Sciences, Beijing 100730, China

## Abstract

**Background:**

Influenza A virus mutates rapidly, rendering antiviral therapies and vaccines directed against virus-encoded targets ineffective. Knowledge of the host factors and molecular pathways exploited by influenza virus will provide further targets for novel antiviral strategies. However, the critical host factors involved in influenza virus infection have not been fully defined.

**Results:**

We demonstrated that LAMP3, a member of lysosome-associated membrane glycoprotein (LAMP) family, was significantly induced in human lung epithelial (A549) cells upon influenza A virus infection. Knockdown of LAMP3 expression by RNA interference attenuated production of viral nucleoprotein (NP) as well as virus titers. Confocal microscopy results demonstrated that viral NP is colocalized within LAMP3 positive vesicles at early stages of virus infection. Furthermore, knockdown of LAMP3 expression led to a reduction in nuclear accumulation of viral NP and impeded virus replication.

**Conclusions:**

LAMP3 is an influenza A virus inducible gene, and plays an important role in viral post-entry steps. Our observations may provide insights into the mechanism of influenza virus replication and potential targets for novel anti-influenza therapeutics.

## Background

Influenza viruses are a significant cause of morbidity and mortality in human as well as avian and animal species worldwide. Antiviral drugs targeting influenza viral proteins, such as oseltamivir, zanamivir, amantadine and rimantadine, have been licensed for treatment of influenza [1]. However, as influenza virus undergoes mutation very rapidly and can evolve drug resistance, the antiviral drugs can become ineffective during a flu outbreak [2,3]. Recently, host factors exploited by influenza virus have attracted increasing interest because therapeutics targeting these cellular factors may inhibit viral replication independent of the antigenic properties of the virus. Therefore, identification of host factors required for viral replication and development of anti-viral agents targeting host factors is a promising strategy for reducing the viral resistance [4-6]. Recently, several research groups have identified a variety of host cell factors that are important for influenza virus replication by using genome-wide RNA interference screenings in conjunction with other integrative genomics strategies [7-11]. These findings provide a global view of the cellular processes that are exploited by influenza viruses and highlighted potential targets which may be used in antiviral research.

The influenza A virus genome is composed of eight segments of negative-sense, single-stranded RNA, which encodes 11 proteins [10]. Hemagglutinin (HA) and neuraminidase (NA) are critical for viral entry and release, respectively, and viral polymerase, composed of three subunits, PA, PB1, and PB2, is responsible for replication and transcription. Following initial interaction of its HA with its N-acetylneuraminic (sialic) acid receptor on the cell surface, the virus enters the cell by receptor-mediated endocytosis. Upon endosomal acidification, the HA protein undergoes conformational changes and mediates fusion between the viral envelope and endosomal membrane. The acidic environment of the endosome also triggers the disassembly of the viral core and the release of the viral ribonucleoprotein (vRNP) into the cytoplasm [12]. The vRNPs are then rapidly imported into the nucleus to catalyze viral genome replication and RNA transcription [13]. Subsequently, newly formed vRNPs, in association with other viral proteins are exported into the cytoplasm and transported to the cell membrane for budding and release. Transcriptional profiling reveals that a variety of host factors were induced upon influenza virus infection [14]. These viral inducible factors may play essential or inhibitory roles in the viral lifecycles, such as viral entry, vRNP trafficking, transcription, viral assembly and budding [9]. However, the function and regulatory mechanisms of these host factors remains largely unknown.

We here report that a host factor, lysosome-associated membrane glycoprotein 3 (LAMP3) is involved in the post-entry stages of influenza A virus infection. We found that LAMP3 was significantly up-regulated upon influenza A virus infection. Knockdown of LAMP3 expression by RNA interference inhibits viral replication in the early stage, suggesting that LAMP3 may play an important role in influenza life cycles.

## Results

### LAMP3 is induced upon influenza A virus infection

**Figure 1:**
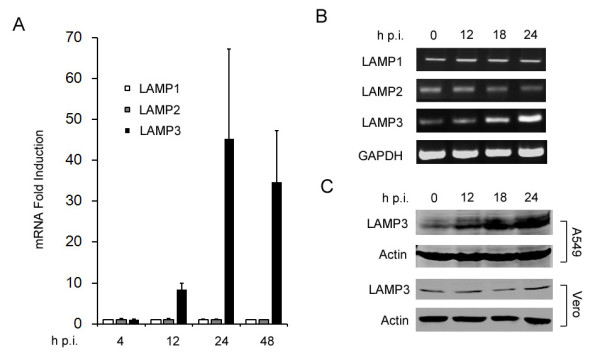
**LAMP3 is specifically induced upon influenza A virus infection**. (A) Microarray results for LAMP1, LAMP2, and LAMP3 expression at different time points post influenza virus infection. A549 cells were infected with A/PR/8/34 virus at a MOI of 0.5. At indicated time points post-infection (p.i.), total cellular RNA was extracted and subjected to microarray analysis. (B) RT-PCR analysis for LAMP1, LAMP2, and LAMP3 expression. A549 cells were infected with A/PR/8/34 virus. After 12 h, 18, or 24 h, total cellular RNA was extracted and subjected to RT-PCR with specific primers. GAPDH gene was used as an internal control. (C) Western blot analysis for LAMP3 expression. A549 or Vero cells were treated as indicated in (B) and were lysed with RIPA buffer. Cell lysates were subjected to Western blot analysis with antibodies against LAMP3. β-actin was used as a loading control.

Influenza A virus infection induces multiple host gene expression. Interestingly, many of the up-regulated host factors appear to function by facilitating virus replication [9]. To investigate the dynamic host gene response profile upon influenza A virus infection, we utilized DNA microarray technology to determine global cellular mRNA levels at different time points post viral infection. Briefly, A549 cells were infected with influenza A/PR/8/34 virus at a multiplicity of infection (MOI) of 0.5. Then, total RNA were prepared at 4 h, 12 h, 24 h, and 48 h post-infection (p.i.), and were subjected to global gene expression analysis by microarray chips (Capitalbio human genome oligo array service, data not shown). Notably, LAMP3, also known as DC-LAMP, TSC403 or CD208 [15], was found to be among the most significantly upregulated genes. Upon A/PR/8/34 virus infection, LAMP3 mRNAs were stimulated a 45.2 fold at 24 h p.i., and a 34.7 fold at 48 h p.i., respectively (Figure 1A). Nevertheless, the mRNA levels of two other LAMP protein family members, LAMP1 and LAMP2, were not altered at any time points indicated, suggesting that LAMP3 may be specifically stimulated by viral infection. To confirm the gene chip data, we used RT-PCR to detect the mRNA level of LAMP1, LAMP2, and LAMP3 at different time points post influenza A virus infection. Consistently, LAMP3, but not LAMP1 and LAMP2, is significantly induced upon influenza A virus infection (Figure 1B). Finally, we determined the protein expression level of LAMP3 in A549 cells and IFN-deficient Vero cells [16], and the results demonstrated that, upon viral infection, LAMP3 protein was upregulated in A549 cells but not in Vero cells, indicating that LAMP3 induction may be interferon-dependent (Figure 1C).

### Knockdown of LAMP3 inhibits influenza A virus replication

**Figure 2:**
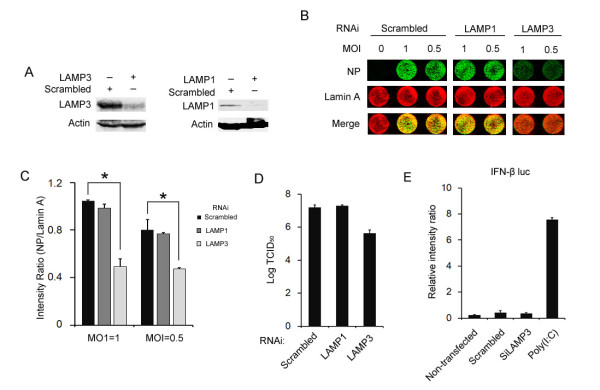
**Knockdown of LAMP3 inhibits influenza A virus replication**. (A) Knockdown of LAMP1 or LAMP3 by RNAi. A549 cells were transfected with the siRNA against LAMP1, LAMP3 or a non-targeting control RNA (scrambled) (50 nM). Seventy two hours later, cells were lysed and subjected to Western blot analysis using antibody against LAMP1 or LAMP3. (B) In-cell western assay for NP production. A549 cells grown in 96-well plates were transfected siRNAs as indicated in (A). After 72 h, cells were either infected or mock infected by influenza virus, and were subjected to in-cell western assay at 24 h p.i.. NP was visualized via labeling with NP specific antibody, and endogenous lamin A was labeled as a control. (C) Quantitative analysis of in-cell western using Odyssey Imaging System. * Student's t test, *p *= 0.031.(D) A549 cells were transfected with LAMP3, LAMP1 or control siRNAs, cells were then infected with A/PR/8/34 virus at a MOI of 0.1 at 72 h post-transfection. Cell supernatants were harvested and subjected to TCID_50 _assay 24 h p. i.(E) A549 cells were left untransfected, or were transfected with an IFN-β reporter plasmid, along with the scrambled siRNA, siRNA against LAMP3 (SiLAMP3), or poly (I:C) at 50 ng/ml (a positive control for IFN promoter activation), and analyzed 48 h later for luciferase assay.

LAMP3 is significantly and specifically induced upon influenza A virus infection, implying that LAMP3 may be involved in virus replication. To address the role of LAMP3 in virus replication, we silenced the expression of LAMP3 or LAMP1 in A549 cells by using specific siRNA oligonucleotides. Total amount of the LAMP3 or LAMP1 protein was detected at 72 h post transfection by Western blot analysis. In cells transfected with LAMP3 or LAMP1 siRNA, protein expression was substantially inhibited (Figure 2A). We next examined if loss of LAMP3 or LAMP1 affects viral protein synthesis. A549 cells were infected withA/PR/8/34 virus at a MOI of 1 or 0.5 after transfection with either scrambled (control) or gene specific siRNA for 72 h. Twenty four hours post viral infection, cells were fixed and stained with anti-NP and anti-lamin A antibody simultaneously for in-cell western analysis [17]. The results showed that the amount of NP production in LAMP3 knockdown cells is remarkably lower than that in control cells and in LAMP1 knockdown cells (Figure 2B), indicating that loss of LAMP3, but not LAMP1, affects virus protein synthesis. Further quantitative analysis for in-cell western results showed that NP production is reduced by nearly 50% in the LAMP3 knockdown cells (Figure 2C). Then, we determined if knockdown of LAMP3 reduces virus titers. Briefly, A549 cells were transfected with LAMP3, LAMP1 or control siRNAs, and cells were infected with A/PR/8/34 virus at a MOI of 0.1 after 72 h. At 24 h p.i., viral supernatants were harvested and subjected to TCID_50 _assay. Consistently, the virus titer was reduced by a 1.5 log10 in LAMP3 knockdown cells compared to the control cells or the LAMP1 knockdown cells (Figure 2D), indicating that LAMP3 plays an important role in the viral replication. Since some siRNAs could induce interferon response, we tested if the inhibition of viral replication by LAMP3 siRNA is related to siRNA induced interferons. The results suggested that transfection of LAMP3 or scrambled siRNAs could not induce IFN-β promoter activity (Figure 2E), indicating that inhibition of viral replication could be attributed to LAMP3 depletion rather than siRNA induced IFN response.

### Influenza virus accumulates in LAMP3 positive vesicles

**Figure 3:**
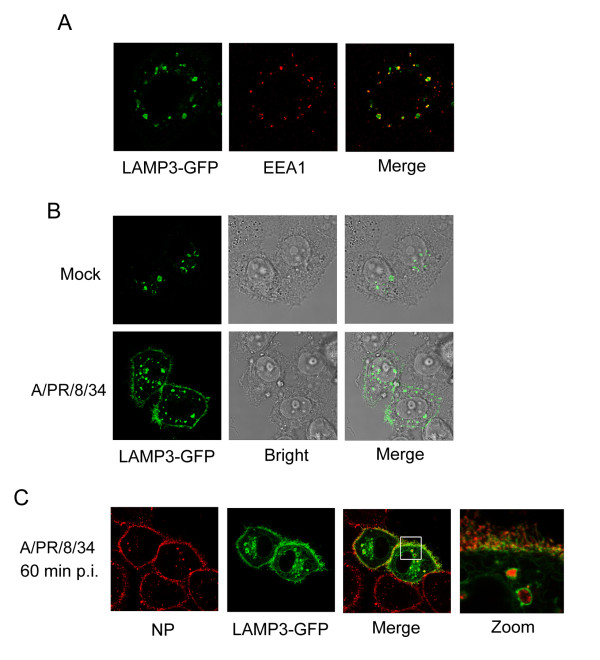
**Influenza virus accumulates in LAMP3 positive vesicles**. (A) Localization of LAMP3 and early endosome marker EEA1. Hela cells were transfected with a LAMP3-GFP plasmid. Eighteen hours later, cells were stained with EEA1 antibody and analyzed by confocal microscopy. (B) LAMP3 translocated to plasma membrane during influenza virus infection. Hela cells transfected with LAMP3-GFP were infected with A/PR/8/34 virus at a MOI of 10 for 1 h. Cells were then fixed and analyzed by confocal microscopy. (C) LAMP3 colocolized with influenza A virus. Hela cells were transfected with LAMP3-GFP for 18 h and then infected with influenza A virus at 10 MOI. After 1 h, cells were stained with the anti-NP antibody and were subjected to confocal microscopy analysis.

Previous studies suggested that LAMP3 is involved in the endosomal/lysosomal pathways [18]. To examine if LAMP3 is involved in influenza A virus trafficking, we determined the localization of LAMP3 and the virus NP by confocal microscopy. First we detected if LAMP3 is localized to the endosome compartments. Hela cells were transfected with GFP tagged LAMP3 and then labeled with EEA1, a marker of early endosomes [19]. Upon merging both channels, the LAMP3-GFP largely colocalize with the EEA1 labeled cytoplasmic compartments of early endosomes (Figure 3A), indicating that LAMP3 may reside in the early endosomes, which is involved in the influenza endocytic pathway. We next determined if the localization of LAMP3 changes upon virus infection. Hela cells transfected with LAMP3-GFP were infected with A/PR/8/34 virus at a MOI of 10. After 1 h, cells were fixed and subjected to confocal microscopy analysis. In the virus infected cells, part of the LAMP3 translocated from the cytoplasm to the plasma membrane (Figure 3B), suggesting that virus infection may regulate LAMP3 trafficking. Finally, we examined if LAMP3 is colocalized with influenza virus. LAMP3-GFP transfected cells were infected with A/PR/8/34 virus at a MOI of 10 for 1 h and stained with antibody against NP. By confocal microscopy, the cytoplasmic speckles of influenza NP colocalized within the LAMP3 positive vesicles (Figure 3C), indicating that LAMP3 may be involved in influenza endocytosis or vRNP trafficking pathways.

### LAMP3 regulates post-entry steps of influenza A virus replication

**Figure 4:**
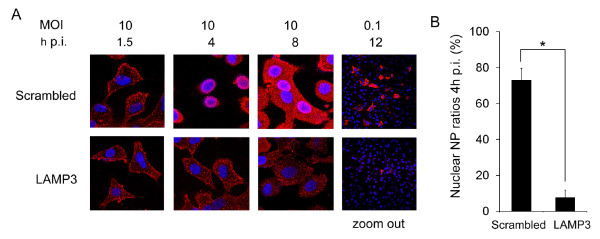
**LAMP3 regulates post-entry steps of influenza A virus replication**. (A) NP localization during influenza A virus replication. A549 cells were transfected with a LAMP3 specific siRNA or a control siRNA (Scrambled), and then infected with A/PR/8/34 virus at a MOI of 10 or 0.1. Cells were fixed at indicated time points respectively, and then subjected to indirect immunofluorescence assays for NP localization. (B) Quantitative analysis for ratios of NP nuclear imported cells/total infected cells. Analyses were carried out for cells infected with A/PR/8/34 virus at a MOI of 10 for 4 h. * Student's t test, *p *= 0.016.

To evaluate which steps of viral life cycles LAMP3 may be involved in, we monitored the localization of the NP during influenza A virus infection. A549 cells were transfected with a LAMP3 specific siRNA or a control siRNA, and then infected with A/PR/8/34 virus at a MOI of 10. Cells were fixed at 1.5 h, 4 h, and 8 h, and then subjected to indirect immunofluorescence assays for detection of NP localization. As indicated in the Figure 4A, at 1.5 h p.i., viral NP were observed internalized in the cytoplasm in both control and LAMP3 knockdown cells, suggesting that depleting LAMP3 may not inhibit virus entry. Intriguingly, at 4 h p.i., about 70% of NP translocated to the cell nuclei in the control cells; whereas in the LAMP3 knockdown cells, NP largely remained in the cytoplasm (Figure 4A and 4B). At 8 h p.i., the NP protein diffused in the control cells but accumulated as cytoplasmic puncta in the LAMP3 depleted cells, suggesting a role of LAMP3 in post-entry steps of influenza virus infection, such as viral uncoating or viral ribonucleoproteins (vRNP) trafficking. Consistent with the in-cell western results, at a MOI of 0.1, the production of NP is substantially decreased in the LAMP3 knockdown cells compared to the control cells (Figure 4A), confirming the role of LAMP3 in virus replication.

## Discussion

Influenza viruses have the ability to exploit or impede host cellular responses to support viral replication, and the interactions between viral and cellular factors determine host susceptibility to influenza infection [14,20]. Novel influenza A virus strains emerge periodically, resulting in global pandemics. Thus, identification of critical host factors for influenza virus replication will provide insights into the development of future anti-flu or broad-spectrum antiviral approaches.

LAMP family proteins have been implicated in the regulation of multiple cellular processes, such as cell growth, adhesion, and metastasis. Both LAMP1 and LAMP2 are located primarily in lysosomes, and shuttle between lysosomes, endosomes, and the plasma membrane [21-27]. LAMP3 was first cloned as a novel human lung-specific gene, and was subsequently found to be overexpressed in malignant cells [28,29]. Recent publications suggest that LAMP3 have metastatic potential in cancers [15]. However, the role of LAMP3 in virus-host interactions has not yet been clarified. Here we showed that the expression of LAMP3 is up-regulated upon influenza virus infection. Further we demonstrated that knockdown of LAMP3 inhibited nuclear accumulation of influenza NP protein at early stages of viral infection. These data indicated a critical role for LAMP3 in influenza virus replication.

LAMP3 bears significant similarity to LAMP1 and LAMP2 [28], and may promote metastasis in the same way as LAMP1 and LAMP2 [15]. Intriguingly, only LAMP3 was significantly up-regulated during influenza virus infection. It is not clear whether the expression of LAMP3 is stimulated by virus replication directly or by virus-induced interferon. Our results indicated that influenza A virus could not induce LAMP3 expression in the IFN-deficient Vero cells. Meanwhile, microarray data mining suggested that LAMP3 was significantly upregulated in interferon treated human blood monocytes [30]; implicating a mechanism by which LAMP3 expression may be regulated by virus-induced interferon.

Our results demonstrated that knockdown of LAMP3 did not affect virus entry while attenuated nuclear accumulation of NP at 4 h post-infection. These results suggest that LAMP3 may play a role in influenza virus post-entry events, such as virus uncoating, cytosolic transport, or nuclear import of viral components. Confocal microscopy analysis suggested that LAMP3 is localized to endosomal compartment, and virus particles are found to be accumulated in the LAMP3 positive vesicles, suggesting that it is more likely that LAMP3 plays a role in early post-entry stages of influenza infection rather than nuclear trafficking of vRNP. Further biochemical experiments, such as investigating vRNP-LAMP3 interaction by immunoprecipitation, will provide insights into the mechanism by which LAMP3 regulates influenza virus infection and replication.

In conclusion, we have identified LAMP3 as a specific host factor that is involved in influenza virus infection at post-entry stages. Further investigations of the roles for LAMP3 in influenza virus infection will provide insight into the network of host-virus interactions that control the early steps of influenza virus replication and new pharmacological targets for anti-viral therapies.

## Methods

### Cell culture and virus infection

The human lung carcinoma cell line A549, human cervical cancer cell line HeLa, Madin Darby Canine kidney cell line MDCK and African green monkey kidney cell line Vero were purchased from American Type Culture Collection (ATCC; Manassas, VA) and were maintained in the Dulbecco's modified Eagle's medium (DMEM; Invitrogen, Carlsbad, CA) supplemented with 10% fetal bovine serum (FBS; HyClone, Logan, UT), 100 U/ml penicillin, and 100 μg/ml streptomycin at 37°C. A influenza A virus strain A/PR/8/34 (H1N1) was used in this study. For virus infection, A549 cells or MDCK cells were inoculated with virus at the indicated multiplicities of infection (MOI). Two hours later, the inoculum was removed, and cells were washed twice with Hank's solution prior to the addition of serum-free DMEM containing 5% BSA and 1 μg/ml TPCK-trypsin (Sigma-Aldrich, St. Louis, MO)[16].

### Reverse transcription PCR

To determine the mRNA expression level of various LAMPs during influenza A virus infection, cells were infected with A/PR/8/34 virus at a MOI of 0.5. At different time points post infection, total cellular RNA was extracted using Trizol reagent (Invitrogen) according to the manufacturer's instructions. RNA samples were treated with DNase I (Pierce, Rockford, IL), and reversely transcribed using a Superscript cDNA synthesis kit (Invitrogen) following the manufacturer's protocols. cDNA samples were subjected to PCR amplification and electrophoresis analysis. The primer sequences were as follows:

LAMP1, 5'-GCGAGCTCCAAAGAAATCAA-3' (forward), 5'-TGGACCTGGGTGCCACTAA-3' (reverse), LAMP2, 5'-CTCTGCGGGGTCATGGTG-3' (forward), 5'-CGCACAGCTCCCAGGACT-3' (reverse) LAMP3, 5'-GCGTCCCTGGCCGTAATT-3' (forward) 5'-TGCTTAGCTGGTTGCTGGA-3' (reverse).

### Western blot analysis

Cells were lysed in RIPA buffer containing 150 mM NaCl, 25 mM Tris (pH 7.4), 1% NP-40, 0.25% sodium deoxycholate, 1 mM EGTA, and 1 mM EDTA with proteinase inhibitor cocktail (Roche, Indianapolis, IN), and total protein concentration was determined by the BCA Protein Assay Reagent Kit (Pierce, Rockford, IL)before separation by SDS-PAGE. Expression of LAMP1 or LAMP3 protein was detected by the rabbit polyclonal anti-LAMP1 antibody (Sigma, L1418) or the rabbit polyclonal anti-LAMP3 antibody (AVIVA, ARP41598_P050), respectively.

### RNA interference

siRNA against LAMP1 (M-013481-01), LAMP3 (M-004716-01), along with the non-targeting control (D-001206-13-05) were purchased from Dharmacon (Boulder, CO). A549 cells were transfected with the siRNAs (50 nM) using Dharmafect1 (Dharmacon) according to the manufacturer's instructions.

### Repoter assays

A549 cells were seeded in 24-well plates at a cell density of 1.5 × 10^5 ^cells per well. The next day, cells were left untransfected, or were transfected with pGL3-IFN-β-Luc and pRL-SV40, along with the scrambled siRNA, siRNA against LAMP3, or poly (I:C) using Lipofectamine 2000 (Invitrogen). At 48 h post transfection, cells were harvested, and cell lysates were subjected to luciferase activity assays (Promega, Madison, WI).

### Indirect immunofluorescence assay

A549 cells either infected or mock infected by influenza A virus were fixed in 4% paraformaldehyde and permeabilized with PBS containing 0.5% Triton X-100 (pH 7.4). Then, the cells were blocked with PBST (PBS containing 0.1% Tween-20; pH 7.4) containing 5% BSA for 50 min at room temperature. Further, the cells were incubated with mouse polyclonal anti-influenza A virus NP antibody (1:500; Millipore, Temecula, CA) at 4°C overnight. After three washes with PBST, cells were incubated with anti-mouse IgG/DyLight594-conjugated antibody (1:500; Zhongshanjinqiao Biotech, Beijing, China) for 1 h, and cell nuclei were labeled with Hoechst dye 33258 (Beyotime, Nantong, China) according to the manufacturer's instructions. For localizing LAMP3 and influenza virus, Hela cells were transfected with LAMP3-GFP (Origene, Rockville, MD), and were stained with NP antibody 18 h post-transfection. After extensive washes with PBST, cells were incubated with anti-mouse IgG/DyLight594-conjugated antibody as indicated above. Fluorescence images were obtained by using a Leica TCS SP5 laser scanning confocal microscope.

### In-cell western assay

The cells infected with influenza A virus or mock infected were immunostained with influenza A virus NP antibody together with a rabbit IgG antibody against Lamin A (1:1000; Sigma-Aldrich) as described above. Subsequently, the cells were washed and stained with goat anti-mouse IgG IRDye 800 antibody (1:500; LI-COR, Lincoln, NE) and goat anti-rabbit IgG IRDye 680 antibody (1:500; LI-COR). Protein expression was quantified using the Odyssey Imaging System. For statistical analysis, integrated intensities of fluorescence in wells were determined using software provided with the imager station (LI-COR). The relative amount of NP protein was obtained by normalizing to the endogenous lamin A.

### TCID_50 _assay

The TCID_50 _assay was performed in MDCK cells according to the protocol recommended by the World Health Organization (http://www.who.int/csr/resources/publications/influenza/whocdscsrncs20025rev.pdf), and data were analyzed according to the Reed and Muench method [31].

## Competing interests

The authors declare that they have no competing interests.

## Authors' contributions

ZZ, JW and TH conceived the study and wrote the manuscript. ZZ, QX, YW, and YY carried out the experiments. All authors read and approved the final manuscript.
